# Cannabis knowledge and health literacy among Thai vocational students: a cross-sectional study following policy liberalization

**DOI:** 10.1186/s42238-026-00467-z

**Published:** 2026-07-13

**Authors:** Jeerisuda Orosram, Charuwan Thanawiroon, Chaisit Soontara, Premsuda Kaewrongkham, Napat Tangsakul, Tawanchai Phohom, Prasittichai Poonphol, Teeraporn Sadira Supapaan

**Affiliations:** 1 Department of Pharmacy Practice, Faculty of Pharmaceutical Sciences, Ubon Ratchathani University, Ubon Ratchathani, Thailand; 2 Department of Pharmaceutical Chemistry and Technology, Faculty of Pharmaceutical Sciences, Ubon Ratchathani University, Ubon Ratchathani, Thailand; 3 Department of Pharmacy, Sunpasitthiprasong Hospital, Ubon Ratchathani, Thailand; 4 Faculty of Pharmaceutical Sciences, Ubon Ratchathani University, Ubon Ratchathani, Thailand; 5 Department of Industrial Engineering, Faculty of Engineering, Ubon Ratchathani University, Ubon Ratchathani, Thailand

**Keywords:** Cannabis, Knowledge, Health literacy, Vocational students, Thailand, Substance use, Cannabis education

## Abstract

**Background:**

Thailand’s decriminalization of cannabis in June 2022 substantially increased adolescent exposure. Cannabis knowledge and health literacy among vocational students remain poorly characterized.

**Methods:**

A cross-sectional online survey was conducted from January to March 2024 among vocational secondary students at one vocational institution in a province in northeastern Thailand. Of 2,656 eligible students, 555 provided valid responses (response rate 20.9%). Two 15-item instruments were used to measure cannabis knowledge and cannabis health literacy. Bivariate analyses and three multivariable regression models examined factors associated with knowledge scores, health literacy scores, and cannabis use experience.

**Results:**

Cannabis knowledge was low (mean 7.23/15, SD 4.43; 55.0% low), as was cannabis health literacy (mean 41.72/75, SD 12.72; 48.8% low). Knowledge level was not significantly associated with health literacy level (χ2[4] = 7.04, *p* = 0.134). In adjusted analyses, prior cannabis education and other substance use were associated with higher knowledge scores, whereas male sex and Year 3 enrollment were associated with lower knowledge scores. Higher health literacy scores were associated with prior cannabis education and family relationship quality in adjusted analyses; male sex showed only a weak adjusted association with lower health literacy. Cannabis use experience was associated with other substance use (OR 4.115), prior cannabis education (OR 3.683), male sex (OR 2.621), Year 2 enrollment (OR 1.733), and family relationship quality (OR 1.558).

**Conclusions:**

Cannabis knowledge and health literacy were predominantly low among Thai vocational secondary students. Factual knowledge alone may not translate into functional cannabis health literacy. School-based interventions should strengthen critical appraisal, communication, self-management, and digital health literacy, with attention to male students, students without prior cannabis education, and those with other substance use experience.

## Introduction

The decriminalization of cannabis in June 2022 in Thailand, making it the first nation to decriminalize the cannabis plant in Asia and thereby leaving it off its list of controlled narcotics, marked a paradigm shift in drug policy with extensive implications on public health (Assanangkornchai et al. 2022; (Kalayasiri, and Boonthae, 2023). Before this historic breakthrough, cannabis was listed as a Schedule 5 narcotic in Thailand by the Narcotic Act since 1979, and medical use has only been allowed since 2019 on a conditional basis (Assanangkornchai et al. 2022). The rapid liberalization of the regulatory background provided an environment where cannabis products were now quite commonly commercially accessible, but the broad-based framework of public health education and the well-defined mechanisms of age-restriction enforcement were not established simultaneously (Kalayasiri, and Boonthae, 2023; Trangenstein et al. 2023).

Adolescent groups are especially vulnerable to the adverse health effects of such a policy shift. The lines of evidence have converged to show that exposure to cannabis in adolescence is correlated with cortical neurodevelopment disruption (Albaugh et al. 2021; Scott 2023), impaired academic performance (Chan et al. 2024), and increased risk of incident psychotic, bipolar, depressive, and anxiety disorders (Young-Wolff et al. 2026). Adding to these risks, it is found that the adolescents who start to use cannabis earlier in life continue to experience more severe and chronic cognitive impairments as compared to those who start to use cannabis during adulthood (Scott 2023; Ross and Bhatt 2023). Strong control of such harms is not only possible by regulation but also by developing evidence-based cannabis information and operational health literacy (HL) among the youth (2022; Harrison et al. 2024).

Health literacy in the cannabis context refers to the ability of an individual to critically assess the information available on cannabis, comprehend the legal and pharmacological situation of cannabis products, communicate effectively with healthcare providers about cannabis, and make informed decisions about using or not using cannabis products (2022; Nutbeam 2008; Sørensen et al. 2012; Leos-Toro et al. 2020). Research undertaken in post-legalization settings, mainly in Canada and the United States, has revealed that the knowledge and health literacy of youth about cannabis is often poorly developed despite the extensive campaigns by the state to create awareness (Harrison et al. 2024; Kourgiantakis et al. 2024).

Vocational secondary students are a highly under-researched but high-priority group in the Thai cannabis health literacy environment. This group of 15–18-year-olds enrolled in applied occupational programs has been reported to have almost twice the lifetime rates of cannabis use compared to peers in general academic education (Poonphol et al. 2026), and has been found to use cannabis as the most frequently used illicit drug by vocational students in Thai urban schools (Poonphol et al. 2026). The distinctive social environments, peer groups, and economic sensitivities that come with vocational educational tracks can increase susceptibility to cannabis usage and obstacles to attaining correct health messages, which may lead to misinformation and a lack of understanding about the risks associated with cannabis use. Education about cannabis beforehand; individual history of substance use; family; and peer relationships might all mediate between institutional learning and knowledge acquisition as well as health literacy gains in this population (Downey et al. 2024; Fleary et al. 2024; González-Cano-Caballero et al. 2023; Torrejón-Guirado et al. 2023). Despite this high-risk profile, limited empirical evidence is available on cannabis knowledge and health literacy specifically among vocational students in Thailand, leaving an important evidence gap that the current study aims to address.

The aim of the current study was to: (1) Characterize cannabis knowledge and cannabis health literacy among Thai vocational secondary students; (2) examine the association between cannabis knowledge level and health literacy level; (3) identify sociodemographic and behavioral factors associated with cannabis knowledge and health literacy scores; and (4) examine factors associated with personal cannabis use experience in this population.

## Methods

### Study design and setting

The survey design used was a cross-sectional study. Convenience sampling was applied to select participants, who were students in one vocational secondary school in a province in northeastern Thailand, included students in Year 1 (around age 15–16), Year 2 (around age 16–17), and Year 3 (around age 17–18). The data were collected in the form of a self-administered structured online questionnaire via Google Forms. The questionnaire had to be completed on a voluntary basis, and the participants were promised anonymity and confidentiality of their answers.

### Participants

The target population comprised 2,656 vocational secondary school students enrolled in Years 1–3 at one vocational institution in a province in northeastern Thailand during the 2024 academic year. The minimum required sample size was calculated using the formula for finite populations: *n* = n₀/[1 + (n₀ − 1)/N], where n₀ = Z^2^p(1 − p)/e^2^, with a 95% confidence level (Z = 1.96), a conservative proportion of *p* = 0.50, a margin of error of e = 0.05, and *N* = 2,656, yielding a minimum of *n* = 336 (Qing and Valliant 2024). The questionnaire link was distributed to all 2,656 eligible students via classroom teachers during scheduled class periods; 555 valid responses were received, yielding a response rate of 20.9%. Participants were recruited using convenience sampling. For students aged under 18 years, parental consent was requested through a mandatory declaration included in the online questionnaire before students could access the survey items. The study was approved by the Ubon Ratchathani University Research Ethics Committee, Thailand (UBU-REC-94/2567).

### Instruments

A 15-item questionnaire assessed cannabis knowledge in four areas: indicators and guidelines of medical cannabis use (K1-K4; maximum 4 points), adverse effects of medical cannabis (K5-K7; maximum 3 points), cannabis-related legislation (K8-K10; maximum 3 points), and cannabis use in food products and regulatory limits. Low (0–8), Moderate (9–12), and High (13–15) scores were assigned. The measures of cannabis health literacy were based on a 15-item, five-point Likert-scale instrument (sum 15–75) adapted from the General Health Literacy Scale developed and validated for Thai populations (Intarakamhang et al. 2022).

The adaptation involved modifying item content from a general health context to cannabis-specific health information, services, and decision-making scenarios, while retaining the original five-domain structure, five-point Likert-scale format, and scoring procedures. Both the original scale and the adapted instrument were developed in Thai; no back-translation was therefore required. Content validity was confirmed by three subject-matter experts using item-objective congruence (IOC) evaluation, and pilot testing was conducted with 30 students prior to full data collection (Cronbach’s α = 0.974). The instrument included five domains: (1) access to health information and services (HL1–HL4; maximum score = 20), (2) comprehension of health information (HL5–HL7; maximum score = 15), (3) appraisal of health information (HL8–HL9; maximum score = 10), (4) communication and social support (HL10–HL12; maximum score = 15), and (5) self-management behaviors (HL13–HL15; maximum score = 15). Total scores were categorized as Low (< 45, i.e., < 60% of maximum), Moderate (45–60, 60–80%), or High (> 60, > 80%). Item-level thresholds were classified as follows: Low (mean < 3.0), Moderate (3.0–4.0), and High (> 4.0).

Cannabis education, experience, other substance use, income, and family and peer relationships were other variables. Content validity was assessed by three subject-matter experts using item-objective congruence (IOC) procedures. Internal consistency was evaluated in a pilot sample of 30 students, yielding Kuder–Richardson Formula 20 (KR-20) = 0.865 for the knowledge scale and Cronbach’s α = 0.974 for the health literacy scale.

### Statistical analysis

Descriptive statistics were computed for all variables, including means, standard deviations, frequencies, and proportions. Group differences in cannabis knowledge and health literacy scores were examined using independent samples t-tests for binary grouping variables. Associations between continuous variables (family and peer relationship quality) and outcome scores were examined using Pearson’s correlation coefficients. Spearman’s rank correlations were used for associations involving ordinal variables and the binary cannabis use experience variable. Chi-square tests of independence were used to examine associations between categorical variables and cannabis use experience. The association between cannabis knowledge level and health literacy level was examined using a chi-square test. Three multivariable regression models were conducted to identify factors independently associated with each outcome while controlling for potential confounders. Multiple linear regression was used for cannabis knowledge scores (Model 1) and health literacy scores (Model 2), with sex, school year (dummy coded: Year 2 vs. Year 1 and Year 3 vs. Year 1, with Year 1 as reference), prior cannabis education, other substance use experience, family relationship quality, and peer relationship quality entered simultaneously. Binary logistic regression was used for cannabis use experience (Model 3) with the same variables. Bivariate analyses used the full sample where applicable (*n* = 555). For multivariable regression analyses, 11 participants who declined to specify sex were excluded because sex was included as a binary predictor, resulting in an analytic sample of *n* = 544. Unstandardized regression coefficients (B), odds ratios (OR), 95% confidence intervals (CI), and effect sizes (Cohen’s d for t-tests; Cramer’s V for chi-square tests) are reported. Statistical significance was set at a two-sided α of 0.05. Exact *p*-values are reported where appropriate, with values below 0.001 reported as *p* < 0.001. All analyses were conducted using Python 3.12, with SciPy used for inferential tests and Statsmodels used for regression analyses. Multicollinearity among variables was assessed using variance inflation factors; all VIF values were below 1.5, indicating no multicollinearity concern.

## Results

### General characteristics of participants

The study included 555 vocational secondary school students. Table 1 summarizes sample characteristics. Most participants were men (*n* = 295; 53.2%). The average age was 16.31 (SD = 1.02). All participants attended vocational schools. Most of the students were year 1 students (*n* = 341, 61.4%).

**Table 1:** General characteristics of participants (*n* = 555)

Characteristic	n	%
1. Sex		
Male	295	53.2
Female	249	44.9
Prefer not to specify	11	2.0
2. Age (mean = 16.31 years; SD = 1.02)		
≤ 15 years	116	20.9
16 years	225	40.5
17 years	156	28.1
≥ 18 years	58	10.5
3. Current Level of Education		
Year 1	341	61.4
Year 2	158	28.5
Year 3	56	10.1
4. Source of Income		
Parental support	501	90.3
Self-funded	51	9.2
Other	3	0.5
5. Monthly Personal Expenses (THB)		
< 3,000 (< approximately USD 94)	253	45.6
3,000–5,999 (approximately USD 94–187)	216	38.9
6,000–9,999 (approximately USD 187–312)	46	8.3
10,000–15,000 (approximately USD 312–468)	29	5.2
> 15,000 (> approximately USD 468)	11	2.0
6. Prior Cannabis Education (formal training/coursework)		
Yes	342	61.6
No	213	38.4
7. Family Relationship Quality (1–5; mean = 4.14; SD = 0.88)		
Level 1 (lowest)	8	1.4
Level 2	14	2.5
Level 3	91	16.4
Level 4	219	39.5
Level 5 (highest)	223	40.2
8. Peer Relationship Quality (1–5; mean = 4.06; SD = 0.87)		
Level 1 (lowest)	8	1.4
Level 2	10	1.8
Level 3	118	21.3
Level 4	223	40.2
Level 5 (highest)	196	35.3
9. Cannabis Use Experience		
Ever used cannabis (any form)	94	16.9
Never used cannabis	461	83.1
Type of cannabis use		
Medical cannabis	24	4.3*
Cannabis in food/beverages	66	11.9*
Recreational cannabis	59	10.6*
Adverse effects from cannabis use	28	5.0*
10. Other Substance Use Experience		
Ever used other substance(s)	264	47.6
Never used other substances	291	52.4
Type of substance (multiple responses permitted)		
Tobacco/e-cigarettes	118	21.3†
Alcoholic beverages	178	32.1†
Kratom	145	26.1†

^*^Percentages for cannabis use types and adverse effects were calculated using the total sample as the denominator (*n* = 555); multiple responses were permitted among students who had ever used cannabis. † Percentages calculated from total sample (*n* = 555); multiple responses permitted

Ninety-four respondents (16.9%) reported using cannabis. Cannabis was most commonly used in food or beverages (*n* = 66, 11.9%) followed by recreational (*n* = 59, 10.6%) and medical (*n* = 24, 4.3%) use. 28 of 94 ever-users (29.8%; 5.0% of the total sample) reported cannabis-related side effects.

### Cannabis knowledge and health literacy levels

Table 2 shows the general and domain-specific cannabis knowledge and health literacy scores. The average general cannabis knowledge was 7.23 (SD = 4.43), which is 48.2 percent of the total possible 15, and was rated as low. The four knowledge areas were also rated low with 43.6 to 59.9% of their highest scores.

**Table 2:** Cannabis knowledge and health literacy scores (*n* = 555)

Section A — Cannabis Knowledge (15 items; maximum score = 15)				
Domain	**Max Score**	**Mean (SD)**	**% of Max Score**	**Level**
Indications & guidelines for medical cannabis use (K1–K4)	4	1.75 (1.13)	43.8%	Low
Adverse effects of medical cannabis (K5–K7)	3	1.80 (1.27)	59.9%	Low
Cannabis-related legislation (K8–K10)	3	1.50 (1.01)	50.2%	Low
Cannabis use in food products and regulatory limits (K11–K15)	5	2.18 (1.92)	43.6%	Low
Overall knowledge score	**15**	**7.23 (4.43)**	**48.2%**	**Low**
Section B — Cannabis Health Literacy (15 items; max score = 75; Likert scale 1–5)				
Domain	**Items (Max Score)**	**Mean (SD)**	**% of Max Score**	**Level**
Access to health information and services (HL1–HL4)	4 (20)	11.20 (3.62)	56.0%	Low
Comprehension of health information (HL5–HL7)	3 (15)	8.62 (2.87)	57.5%	Low
Appraisal of health information (HL8–HL9)	2 (10)	5.62 (1.92)	56.2%	Low
Communication and social support (HL10–HL12)	3 (15)	8.22 (2.76)	54.8%	Low
Self-management behaviors (HL13–HL15)	3 (15)	8.06 (2.85)	53.7%	Low
Overall health literacy score	**15 (75)**	**41.72 (12.72)**	**55.6%**	**Low**
Section C — Level Distribution Summary				
Outcome	**Low**	**Moderate**	**High**	**Total**
Cannabis Knowledge	305 (55.0%)	168 (30.3%)	82 (14.8%)	555
Cannabis Health Literacy	271 (48.8%)	258 (46.5%)	26 (4.7%)	555

### Cannabis knowledge by specific items

Table 3 presents item-level response frequencies for all 15 cannabis knowledge questions. Correct response rates varied considerably across items, revealing specific areas of particular knowledge deficit. In Domain 1, K1, which measured the level of understanding that cannabis could be used to prevent nausea and vomiting caused by chemotherapy, had the lowest correct response rate in the domain at 29.9%, and 70.1% of the respondents had incorrect responses or were not sure.

**Table 3:** Cannabis knowledge by specific items (*n* = 555)

Item	Question	Correct, n (%)	Incorrect/Uncertain, n (%)
Domain 1: Indications & Guidelines for Medical Cannabis Use (K1–K4)			
K1	Cannabis can alleviate nausea and vomiting induced by chemotherapy	166 (29.9%)	389 (70.1%)
K2	Cannabis can treat all diseases (e.g., nausea, seizures, spasticity).*	127 (22.9%)	428 (77.1%)
K3	Cannabis use is prohibited in pregnant and breastfeeding women	385 (69.4%)	170 (30.6%)
K4	Patients with schizophrenia should consult a physician or pharmacist before using cannabis	294 (53.0%)	261 (47.0%)
Domain 2: Adverse Effects of Medical Cannabis (K5–K7)			
K5	A side effect of cannabis is dizziness	332 (59.8%)	223 (40.2%)
K6	A side effect of cannabis is hallucinations	355 (64.0%)	200 (36.0%)
K7	A side effect of cannabis is rapid heartbeat	311 (56.0%)	244 (44.0%)
Domain 3: Cannabis-Related Legislation (K8–K10)			
K8	Cannabis use is prohibited in public places	411 (74.1%)	144 (25.9%)
K9	Cannabis extract containing ≤ 0.2% THC by weight is not classified as a Schedule 5 narcotic	182 (32.8%)	373 (67.2%)
K10	Individuals over 15 years may possess, use, transport, and sell cannabis.*	242 (43.6%)	313 (56.4%)
Domain 4: Cannabis Use in Food Products and Regulatory Limits (K11–K15)			
K11	Using cannabis flowers in food preparation for commercial sale is prohibited	268 (48.3%)	287 (51.7%)
K12	Mixing cannabis with alcohol for commercial sale is prohibited	310 (55.9%)	245 (44.1%)
K13	Mixing cannabis with caffeine for commercial sale is prohibited	303 (54.6%)	252 (45.4%)
K14	Tetrahydrocannabinol (THC) is an addictive substance	195 (35.1%)	360 (64.9%)
K15	Cannabidiol (CBD) is a non-addictive compound	134 (24.1%)	421 (75.9%)

^*^Items marked with an asterisk are false statements; a correct response is therefore 'Incorrect'

### Cannabis health literacy by specific items

Table 4 shows the mean scores and standard deviation of all 15 cannabis health literacy statements by item and based on the revised five-domain framework. Item-level mean scores ranged from 2.65 to 2.90, with all 15 items classified as Low (mean < 3.0) on a five-point Likert scale, so all the statements fell in the low literacy category.

**Table 4:** Health literacy regarding cannabis use by specific items (*n* = 555)

Item	Statement	Mean	SD	Level
Domain 1: Access to Health Information and Services (HL1–HL4; max = 20)				
HL1	I can search for accurate cannabis-related information from multiple sources	2.87	1.03	Low
HL2	I always access cannabis-related health information from credible sources to maintain good health	2.77	1.07	Low
HL3	I can independently seek out health information related to cannabis	2.86	1.04	Low
HL4	I can conveniently consult healthcare professionals regarding cannabis use	2.70	1.02	Low
Domain 2: Comprehension of Health Information (HL5–HL7; max = 15)				
HL5	I can correctly follow instructions provided in cannabis use manuals or documents	2.84	1.06	Low
HL6	I can understand cannabis-related information from various media sources	2.90	1.04	Low
HL7	I can understand explanations or recommendations provided by healthcare professionals regarding cannabis use	2.89	1.05	Low
Domain 3: Appraisal of Health Information (HL8–HL9; max = 10)				
HL8	I compare cannabis information from multiple sources to verify accuracy before sharing it with others	2.82	1.04	Low
HL9	I consult healthcare professionals about health-related practices concerning cannabis before following them	2.80	1.02	Low
Domain 4: Communication and Social Support (HL10–HL12; max = 15)				
HL10	I have relatives or friends who can advise or assist me regarding cannabis use and health	2.70	1.03	Low
HL11	I can ask healthcare professionals questions I want to know about cannabis use	2.82	1.03	Low
HL12	I can appropriately convey cannabis-related health information to others	2.71	1.04	Low
Domain 5: Self-Management Behaviors (HL13–HL15; max = 15)				
HL13	I have health knowledge about cannabis to appropriately take care of my own health	2.65	1.03	Low
HL14	I can adjust my cannabis-related behaviors in accordance with health information received	2.69	1.07	Low
HL15	I routinely monitor changes or abnormalities in my body and mind if I need to use cannabis	2.71	1.06	Low

Response scale: 1 = least to 5 = most. Level per item mean: Low < 3.0; Moderate 3.0–4.0; High > 4.0

### Association between cannabis knowledge level and health literacy level

Table 5 presents the cross-tabulation of cannabis knowledge level by cannabis health literacy level. Among participants with low cannabis knowledge (*n* = 305), 51.8% had low health literacy and 44.3% had moderate health literacy. Among those with high cannabis knowledge (*n* = 82), 58.5% had moderate health literacy compared with participants in lower knowledge categories.

**Table 5:** Association between cannabis knowledge level and health literacy level (*n* = 555)

Knowledge Level	HL: Low n (%)	HL: Moderate n (%)	HL: High n (%)	Row Total n (%)
Knowledge: Low	158 (51.8%)	135 (44.3%)	12 (3.9%)	305 (55.0%)
Knowledge: Moderate	83 (49.4%)	75 (44.6%)	10 (6.0%)	168 (30.3%)
Knowledge: High	30 (36.6%)	48 (58.5%)	4 (4.9%)	82 (14.8%)
Column Total	271 (48.8%)	258 (46.5%)	26 (4.7%)	555 (100.0%)

χ^2^(4) = 7.04, *p* = 0.134. No statistically significant association between knowledge level and health literacy level (*p* > 0.05)

### Sociodemographic and behavioral factors associated with cannabis knowledge

Table 6 presents the t-tests and Pearson correlations for cannabis knowledge scores. Female students had significantly higher knowledge scores than male students (7.71 [SD = 4.22] vs. 6.79 [SD = 4.59]; t = − 2.42, *p* = 0.016; Cohen’s d = 0.21). Prior cannabis education was significantly associated with higher knowledge scores (Yes: 7.70 [SD = 4.27] vs. No: 6.49 [SD = 4.58]; t = 3.14, *p* = 0.002; Cohen’s d = 0.27). Cannabis use experience (t = 2.03, *p* = 0.043; Cohen’s d = 0.23) and other substance use experience (Yes: 7.83 [SD = 4.21] vs. No: 6.70 [SD = 4.56]; t = 3.02, *p* = 0.003; Cohen’s d = 0.26) were also significantly associated with higher knowledge scores. All effect sizes were small (Cohen’s d ≤ 0.27). Family relationship quality (r = 0.023, *p* = 0.585) and peer relationship quality (r = 0.039, *p* = 0.357) were not significantly correlated with knowledge scores.

**Table 6:** Sociodemographic and behavioral factors associated with cannabis knowledge score (*n* = 555)

Variable/Group	Group 1 Mean (SD)	Group 2 Mean (SD)	t-statistic	*p*-value
Sex	Male (*n* = 295): 6.79 (4.59)	Female (*n* = 249): 7.71 (4.22)	− 2.42	0.016*
Education level (Year 2 vs Year 1)	Year 2 (*n* = 158): 7.04 (4.68)	Year 1 (*n* = 341): 7.47 (4.33)	− 1.01	0.314
Education level (Year 3 vs Year 1)	Year 3 (*n* = 56): 6.36 (4.18)	Year 1 (*n* = 341): 7.47 (4.33)	− 1.79	0.075
Prior cannabis education	Yes (*n* = 342): 7.70 (4.27)	No (*n* = 213): 6.49 (4.58)	3.14	0.002**
Cannabis use experience	Yes (*n* = 94): 8.07 (4.53)	No (*n* = 461): 7.06 (4.39)	2.03	0.043*
Other substance use experience	Yes (*n* = 264): 7.83 (4.21)	No (*n* = 291): 6.70 (4.56)	3.02	0.003**
Income source	Self (*n* = 51): 6.12 (4.72)	Parent (*n* = 501): 7.34 (4.40)	− 1.87	0.062
Pearson Correlation with Knowledge Score (continuous predictors)				
Variable	**Pearson r**		***p*-value**	
Family relationship quality (1–5)	0.023		0.585	
Peer relationship quality (1–5)	0.039		0.357	

^*^p < 0.05; **p < 0.01

### Sociodemographic and behavioral factors associated with cannabis health literacy

The results of independent samples t-tests and Pearson correlations for cannabis health literacy scores are shown in Table 7. No significant difference in health literacy scores was found between male and female students (male: 40.64 [SD = 12.60] vs. female: 42.68 [SD = 12.55]; t = − 1.89, *p* = 0.059). Adjusted findings are reported in the multivariable regression section. Students in Year 3 had significantly higher health literacy scores than Year 1 students (44.50 [SD = 10.60] vs. 40.76 [SD = 12.93]; t = − 2.05, *p* = 0.041; Cohen’s d = 0.31). Prior cannabis education was significantly associated with higher health literacy scores (Yes: 43.21 [SD = 12.56] vs. No: 39.34 [SD = 12.63]; t = 3.52, *p* < 0.001; Cohen’s d = 0.31). Family relationship quality was positively correlated with health literacy (r = 0.115, *p* = 0.006), while peer relationship quality was not (r = 0.048, *p* = 0.255).

**Table 7:** Sociodemographic and behavioral factors associated with cannabis health literacy score (*n* = 555)

Variable/Group	Group 1 Mean (SD)	Group 2 Mean (SD)	t-statistic	*p*-value
Sex	Male (*n* = 295): 40.64 (12.60)	Female (*n* = 249): 42.68 (12.55)	− 1.89	0.059
Education level (Year 2 vs Year 1)	Year 2 (*n* = 158): 42.82 (12.78)	Year 1 (*n* = 341): 40.76 (12.93)	− 1.66	0.098
Education level (Year 3 vs Year 1)	Year 3 (*n* = 56): 44.50 (10.60)	Year 1 (*n* = 341): 40.76 (12.93)	− 2.05	0.041*
Prior cannabis education	Yes (*n* = 342): 43.21 (12.56)	No (*n* = 213): 39.34 (12.63)	3.52	< 0.001***
Cannabis use experience	Yes (*n* = 94): 41.05 (13.12)	No (*n* = 461): 41.86 (12.64)	− 0.56	0.575
Other substance use experience	Yes (*n* = 264): 41.86 (12.73)	No (*n* = 291): 41.60 (12.72)	0.25	0.803
Income source	Self (*n* = 51): 42.76 (13.61)	Parent (*n* = 501): 41.57 (12.58)	0.64	0.523
Pearson Correlation with Health Literacy Score (continuous predictors)				
Variable	**Pearson r**		***p*-value**	
Family relationship quality (1–5)	0.115		0.006**	
Peer relationship quality (1–5)	0.048		0.255	

^*^p < 0.05; **p < 0.01

### Sociodemographic and behavioral factors associated with cannabis use experience

Table 8 reports chi-square analyses for cannabis use experience. Male students reported a substantially higher prevalence of ever having used cannabis (22.4%) compared with female students (9.6%), a difference that was statistically significant (χ2[2] = 18.57, *p* < 0.001; Cramer’s V = 0.183). Among students who had received prior cannabis education, 22.2% reported having ever used cannabis, compared with 8.5% of those without prior education (χ2[1] = 16.73, *p* < 0.001; Cramer’s V = 0.174). The strongest association was observed for other substance use; 26.9% of students with other substance use experience had also used cannabis, compared with 7.9% of those without (χ2[1] = 34.15, *p* < 0.001; Cramer’s V = 0.248). Income source was not significantly associated with cannabis use experience (χ2[2] = 2.30, *p* = 0.512). Spearman rank correlations indicated that neither family relationship quality (rs = 0.051, *p* = 0.226) nor peer relationship quality (rs = 0.022, *p* = 0.599) was significantly associated with cannabis use experience.

**Table 8:** Sociodemographic and behavioral factors associated with cannabis use experience (*n* = 555)

Variable	Ever Used n (%)	Never Used n (%)	χ^2^ (df)	*p*-value
Sex			18.57 (2)	< 0.001***
Male (*n* = 295)	66 (22.4%)	229 (77.6%)		
Female (*n* = 249)	24 (9.6%)	225 (90.4%)		
Not stated (*n* = 11)	4 (36.4%)	7 (63.6%)		
Education level			11.41 (2)	0.003**
Year 1 (*n* = 341)	48 (14.1%)	293 (85.9%)		
Year 2 (*n* = 158)	40 (25.3%)	118 (74.7%)		
Year 3 (*n* = 56)	6 (10.7%)	50 (89.3%)		
Prior cannabis education			16.73 (1)	< 0.001***
Yes (*n* = 342)	76 (22.2%)	266 (77.8%)		
No (*n* = 213)	18 (8.5%)	195 (91.5%)		
Other substance use			34.15 (1)	< 0.001***
Yes (*n* = 264)	71 (26.9%)	193 (73.1%)		
No (*n* = 291)	23 (7.9%)	268 (92.1%)		
Income source			2.30 (2)	0.512
Self (*n* = 51)	12 (23.5%)	39 (76.5%)		
Parent (*n* = 501)	82 (16.4%)	419 (83.6%)		
Other (*n* = 3)	0 (0.0%)	3 (100.0%)		
Spearman Correlation with Cannabis Use Experience (continuous predictors)	**Ever Mean (SD)**	**Never Mean (SD)**	**Spearman’s rank correlation coefficient (r_s)**	***p*-value**
Family relationship quality (1–5)	4.26 (0.79)	4.12 (0.90)	0.051	0.226
Peer relationship quality (1–5)	4.12 (0.81)	4.05 (0.89)	0.022	0.599

^**^p < 0.01; ***p < 0.001. For continuous predictors, Spearman’s rank correlation (rs) was applied

### Multivariable regression analyses: factors independently associated with cannabis knowledge, health literacy, and cannabis use experience

To identify factors independently associated with each outcome while controlling for potential confounders, three multivariable regression models were conducted (*n* = 544; 11 participants who declined to specify sex were excluded). Tables 9, 10, and 11 present the results of multiple linear regression for cannabis knowledge (Model 1) and health literacy (Model 2), and binary logistic regression for cannabis use experience (Model 3).

**Table 9:** Factors independently associated with cannabis knowledge score: multiple linear regression (Model 1; *n* = 544)

Variable	B	SE	95% CI	*p*-value	Sig
Sex (male = 1)	− 0.986	0.389	[− 1.750, − 0.222]	0.012	*
Year 2 vs. Year 1 (ref.)	− 0.248	0.436	[− 1.105, 0.608]	0.570	NS
Year 3 vs. Year 1 (ref.)	− 1.311	0.643	[− 2.574, − 0.048]	0.042	*
Prior cannabis education	+ 0.977	0.391	[0.210, 1.744]	0.013	*
Other substance use	+ 1.080	0.381	[0.333, 1.828]	0.005	**
Family relationship quality	+ 0.284	0.235	[− 0.177, 0.746]	0.226	NS
Peer relationship quality	+ 0.153	0.232	[− 0.303, 0.609]	0.509	NS

R^2^ = 0.050; Adj.R^2^ = 0.037; F(7,536) = 4.01, *p* < 0.001. B = unstandardized regression coefficient; SE = standard error; CI = confidence interval

^*^p < 0.05; **p < 0.01; NS = not significant. Year 1 = reference category for school year dummy variables. Prior cannabis education and other substance use coded: 1 = yes, 0 = no. *n* = 544 (11 participants who declined to specify sex excluded)

**Table 10:** Factors independently associated with cannabis health literacy score: multiple linear regression (Model 2; *n* = 544)

Variable	B	SE	95% CI	*p*-value	Sig
Sex (male = 1)	− 2.178	1.102	[− 4.344, − 0.013]	0.049	*
Year 2 vs. Year 1 (ref.)	+ 2.114	1.236	[− 0.314, 4.542]	0.088	NS
Year 3 vs. Year 1 (ref.)	+ 2.705	1.823	[− 0.877, 6.286]	0.139	NS
Prior cannabis education	+ 4.178	1.107	[2.003, 6.353]	< 0.001	***
Other substance use	+ 0.331	1.079	[− 1.788, 2.450]	0.759	NS
Family relationship quality	+ 1.482	0.666	[0.175, 2.790]	0.026	*
Peer relationship quality	+ 0.198	0.658	[− 1.091, 1.492]	0.763	NS

R^2^ = 0.052; Adj.R^2^ = 0.039; F(7,536) = 4.18, *p* < 0.001. B = unstandardized regression coefficient; SE = standard error; CI = confidence interval

^*^p < 0.05; ***p < 0.001; NS = not significant. Year 1 = reference category for school year dummy variables. *n* = 544

**Table 11:** Factors independently associated with cannabis use experience: binary logistic regression (Model 3; *n* = 544)

Variable	B	OR	95% CI (OR)	*p*-value	Sig
Sex (male = 1)	+ 0.964	2.621	[1.515, 4.535]	< 0.001	***
Year 2 vs. Year 1 (ref.)	+ 0.550	1.733	[1.015, 2.958]	0.044	*
Year 3 vs. Year 1 (ref.)	− 0.420	0.657	[0.254, 1.699]	0.386	NS
Prior cannabis education	+ 1.304	3.683	[2.023, 6.706]	< 0.001	***
Other substance use	+ 1.415	4.115	[2.391, 7.083]	< 0.001	***
Family relationship quality	+ 0.443	1.558	[1.104, 2.199]	0.012	**
Peer relationship quality	− 0.156	0.856	[0.619, 1.184]	0.347	NS

Pseudo R^2^ (McFadden) = 0.164; LR χ^2^ = 79.86, *p* < 0.001. B = unstandardized logistic regression coefficient; OR = odds ratio; CI = confidence interval

^*^p < 0.05; **p < 0.01; ***p < 0.001; NS = not significant. Outcome: 1 = ever used cannabis, 0 = never used. Year 1 = reference category. *n* = 544

Model 1 (Table 9) explained 5.0% of the variance in cannabis knowledge scores (R^2^ = 0.050, Adj.R^2^ = 0.037; F[7,536] = 4.01, *p* < 0.001). After adjusting for all other variables, male sex was independently associated with lower knowledge scores (B = − 0.986, 95% CI [− 1.750, − 0.222], *p* = 0.012). Prior cannabis education (B = + 0.977, 95% CI [0.210, 1.744], *p* = 0.013) and other substance use experience (B = + 1.080, 95% CI [0.333, 1.828], *p* = 0.005) were each independently associated with higher knowledge scores. After adjustment, Year 3 students had lower knowledge scores than Year 1 students (B = − 1.311, 95% CI [− 2.574, − 0.048], *p* = 0.042), although the corresponding bivariate comparison in Table 6 did not reach statistical significance (*p* = 0.075). Year 2 students did not differ significantly from Year 1 students (*p* = 0.570). Family relationship quality and peer relationship quality were not independently associated with knowledge scores (all *p* > 0.05).

Model 2 (Table 10) explained 5.2% of the variance in cannabis health literacy scores (R^2^ = 0.052, Adj.R^2^ = 0.039; F[7,536] = 4.18, *p* < 0.001). After adjustment, prior cannabis education was the strongest factor independently associated with higher health literacy (B = + 4.178, 95% CI [2.003, 6.353], *p* < 0.001). Family relationship quality was also independently associated with health literacy (B = + 1.482, 95% CI [0.175, 2.790], *p* = 0.026). Male sex showed a weak adjusted association with lower health literacy scores (B = − 2.178, 95% CI [− 4.344, − 0.013], *p* = 0.049). Neither school year (Year 2: *p* = 0.088; Year 3: *p* = 0.139), other substance use, nor peer relationship quality was independently associated with health literacy (all *p* > 0.05).

Model 3 (Table 11) identified several factors independently associated with cannabis use experience (Pseudo R^2^ [McFadden] = 0.164; LR χ^2^ = 79.86, *p* < 0.001). Other substance use experience had the strongest association (OR = 4.115, 95% CI [2.391, 7.083], *p* < 0.001), followed by prior cannabis education (OR = 3.683, 95% CI [2.023, 6.706], *p* < 0.001), and male sex (OR = 2.621, 95% CI [1.515, 4.535], *p* < 0.001). Using dummy coding with Year 1 as reference, Year 2 was independently associated with higher odds of cannabis use experience (OR = 1.733, 95% CI [1.015, 2.958], *p* = 0.044), while Year 3 was not (*p* = 0.386) — consistent with the non-linear pattern observed in bivariate analysis (Year 1: 14.1%, Year 2: 25.3%, Year 3: 10.7%). Family relationship quality was independently associated with cannabis use experience (OR = 1.558, 95% CI [1.104, 2.199], *p* = 0.012), a finding not apparent in bivariate analysis. School year (Year 3) and peer relationship quality were not independently associated with cannabis use experience (all *p* > 0.05).

## Discussion

Cannabis knowledge and health literacy were predominantly low among Thai vocational secondary school students, with fewer than 15% achieving high knowledge and fewer than 5% achieving high health literacy, findings consistent with youth populations in other post-liberalization jurisdictions (Harrison et al. 2024; Leos-Toro et al. 2020) and extending this evidence base to the understudied vocational sector of Southeast Asia.

Item-level analysis identified the most pronounced deficits in areas of direct public health relevance. Correct response rates were low for knowledge that CBD is non-addictive (K15: 24.1%), that THC is addictive (K14: 35.1%), the regulatory THC threshold of 0.2% (K9: 32.8%), and cannabis’ role in alleviating chemotherapy-induced nausea (K1: 29.9%). These gaps carry direct public health consequences: misattribution of addictive potential to CBD may deter legitimate therapeutic use, while inadequate regulatory knowledge may limit consumers’ ability from distinguishing legal from illicit products (Harrison et al. 2024; Poonphol et al. 2026). A parallel Thai study similarly found that over 60% of community hospital outpatients answered ‘do not know’ regarding the therapeutic benefits or adverse effects of cannabis (Gozzoli et al. 2025), suggesting that similar knowledge gaps may also occur in other Thai populations rather than being specific to vocational students.

The absence of a statistically significant association between cannabis knowledge level and health literacy level (χ2[4] = 7.04, *p* = 0.134) is theoretically important. This finding should not be interpreted as evidence of true construct independence, but rather as an absence of observable association under the current bivariate analytical framework. It is consistent with the multidimensional model of health literacy (Sørensen et al. 2012) and Nutbeam’s (Nutbeam 2008) functional–interactive–critical framework: factual cannabis knowledge does not automatically translate into the higher-order competencies required for protective health decision-making. Accordingly, educational interventions must extend beyond information transfer to explicitly cultivate critical appraisal, communication, and self-management skills (Downey et al. 2024).

In bivariate analysis, prior cannabis education was associated with higher knowledge (*p* = 0.002) and health literacy (*p* < 0.001) scores, consistent with Canadian research linking school-based prevention exposure to improved cannabis literacy outcomes (2022; Kourgiantakis et al. 2024).

This association was maintained in multivariable analysis, with prior cannabis education independently associated with knowledge (B = + 0.977, *p* = 0.013; Table 9) and was most strongly associated with health literacy (B = + 4.178, *p* < 0.001; Table 10). However, prior cannabis education was also independently associated with higher odds of cannabis use experience (OR = 3.683, *p* < 0.001; Table 11), suggesting that it may reflect either structured educational exposure or reactive exposure following cannabis-related experience; the causal direction cannot be inferred from the cross-sectional design.

Female students had significantly higher cannabis knowledge scores than male students in bivariate analysis (*p* = 0.016), and male sex remained independently associated with lower knowledge scores after adjustment (B = − 0.986, *p* = 0.012; Table 9). Taken together, these findings suggest that male students constitute an important priority subgroup because higher cannabis use experience co-occurred with lower cannabis knowledge in this group. This pattern may indicate that experiential familiarity with cannabis does not necessarily correspond to accurate risk knowledge or formal cannabis education (Harrison et al. 2024). Consistent with prior evidence on cannabis normalisation and risk perception among adolescent males (Leos-Toro et al. 2020), these findings support the need for targeted, gender-sensitive cannabis education that addresses both factual knowledge and risk appraisal. The differential pattern of associations involving family relationship quality is theoretically instructive. In bivariate analysis, family quality was positively associated with health literacy (r = 0.115, *p* = 0.006) but not with cannabis use experience (rs = 0.051, *p* = 0.226). In multivariable analysis, family relationship quality was independently associated with health literacy (B = + 1.482, *p* = 0.026; Table 10) and cannabis use experience (OR = 1.558, *p* = 0.012; Table 11). This pattern suggests that the positive association between family relationship quality and cannabis use experience may reflect greater family communication and disclosure, unmeasured confounding (such as family attitudes toward cannabis), or other pathways not captured in this cross-sectional design; it does not imply that higher family relationship quality directly increases cannabis use. The association with health literacy is more readily interpretable as reflecting the role of supportive home environments in developing health information skills. Peer relationship quality was not independently associated with any outcome in either bivariate or multivariable analyses (all *p* > 0.05), indicating that global peer relational quality does not independently account for variation in knowledge, health literacy, or cannabis use in this sample. These findings support incorporating family engagement components in school-based interventions targeting both health literacy development and cannabis use prevention (2022; Fleary et al. 2024).

Other substance use had the strongest bivariate association with cannabis use experience (26.9% vs. 7.9%; χ2[1] = 34.15, *p* < 0.001; Cramer’s V = 0.248), and showed the largest adjusted association in logistic regression (OR = 4.115, *p* < 0.001; Table 11). Alcohol (32.1%), kratom (26.1%), and tobacco or e-cigarettes (21.3%) were the most commonly co-used substances. Co-use is associated with compounded risks to academic performance (Chan et al. 2024; Weitzman et al. 2024) and neurodevelopmental trajectories (Scott 2023). Cannabis education programs in Thai vocational settings should therefore be embedded within integrated substance use literacy frameworks rather than delivered as cannabis-specific siloed interventions. The importance of this approach is supported by modeling evidence from Sweden, where decriminalization was projected to produce an immediate increase in both experimental and recurrent cannabis use, with recurrent use continuing to rise over time (Andersson et al. 2026) — a pattern suggesting that policy liberalization should be accompanied by robust health literacy infrastructure to help reduce cannabis-related public health risks. Poonphol et al. (2026) found that 49.2 percent of a mixed general-vocational group (*n* = 252) in northeastern Thailand reported low cannabis knowledge but 62.3 percent moderate health literacy with the same instruments. Its more positive HL profile in that sample may be explained by the fact that it had a mostly general academic composition (79.0%), as in their analysis, vocational students were found to have lower health literacy in comparison with general education counterparts. In both investigations, male students indicated a greater prevalence of cannabis use but lower scores in knowledge—a congruity with the functional division between the risk-seeking and information-seeking behaviors in adolescent males (Ritchwood et al. 2015) and identifying male sex as a consistent priority subgroup for targeted interventions. Also, Poonphol et al. (2026) noted that expressive health literacy competences, critically assessing and sharing cannabis information, were less strong than receptive ones, which reflects the low score (54.8) of the communication domain and self-management (53.7) domain in the current research: school-based interventions should expressly train critical appraisal, peer communication, and informed decision-making (Ritchwood et al. 2015).

These patterns are not confined to adolescents. Thongtaeng and Seesawang (2026) found that older Thai adults with moderate cannabis knowledge continued to rely predominantly on informal online sources for health decisions (Thongtaeng and Seesawang 2026), suggesting a possible gap between cannabis awareness and functional competency across age groups. For vocational adolescents — who are developmentally more susceptible to peer influence and digital misinformation (Torrejón-Guirado et al. 2023)—this disconnect carries heightened risk, underscoring digital and media literacy as priority components of cannabis health education.

The broader behavioral context amplifies these concerns. Post-legalization increases in high-risk cannabis practices among Thai adults — including driving after use—raise concern that inadequate cannabis health literacy may have implications for public safety, not merely education (Jayuphan et al. 2025). Youth cannabis accessibility has also expanded since liberalization, with youth prevalence exceeding the general population (Insiri 2022), situating vocational students as a particularly high-risk sub-population warranting targeted intervention.

The deficits documented here may reflect gaps in education, health communication, and related support infrastructure. Addressing these gaps may require a multi-level response integrating individual skill-building (critical appraisal, communication, self-management), institutional support (curriculum development, educator preparedness), and coordinated public health communication (coherent, digital-era public health messaging). The policy context further underscores the importance of this issue, as Thailand’s cannabis regulatory environment remains dynamic. Longitudinal surveillance of youth cannabis knowledge and health literacy following future regulatory changes would therefore be valuable.

### Limitations

There are several limitations to be considered in regard to interpretation of these findings. First, the use of convenience sampling in a single vocational institution in one province in the northeast of Thailand limits generalizability of the findings to vocational students at a national or regional level. The sample may not be representative of vocational students with different demographic, socioeconomic or institutional characteristics. Second, the cross-sectional design does not allow inference of causal or directional relationships. For example, the association between prior cannabis education and cannabis use experience may be bidirectional which cannot be disentangled without longitudinal data. Third, self-reported measures of cannabis and substance use are susceptible to social desirability bias which could lead to underreporting of use. No biomarker verification or independent validation was available. Fourth, although both instruments demonstrated acceptable content validity (via expert IOC evaluation) and internal consistency (KR-20 = 0.865; Cronbach’s α = 0.974), construct validity has not been confirmed through confirmatory factor analysis or item response theory, and test–retest reliability was not assessed. Fifth, the multivariable regression models explained only 5.0% and 5.2% of the variance in knowledge and health literacy scores respectively, indicating that important predictors not captured in this study — such as media exposure, parental education, or community-level factors — likely account for substantial unexplained variation. Sixth, the study was conducted during a period of highly dynamic cannabis policy in Thailand. The rapid legislative changes between 2022 and 2024 may have differentially influenced participants’ awareness and attitudes, and findings may not reflect the situation following future regulatory amendments.

## Conclusions

Thai vocational secondary school students had a general low level of cannabis knowledge and health literacy. The fact that the association between the two constructs is not significant indicates that factual knowledge might not be enough to translate to functional skills of protective health action. The most consistent factor independently associated with both knowledge and health literacy was prior formal cannabis education, while family relationship quality was independently associated with health literacy. Cannabis use experience was independently associated with male sex, Year 2 enrollment, prior cannabis education, other substance use, and family relationship quality. These results support the need for cannabis health literacy interventions in vocational schools that reinforce critical appraisal, communication, self-management, and digital literacy skills. Further interventions should include family involvement and specific interventions with high-risk populations, such as male students, particularly given their higher cannabis use experience and lower adjusted knowledge scores, students with no prior cannabis education, and students with other substance use experience.

## Data Availability

No datasets were generated or analysed during the current study.

## Abbreviations

CBD: Cannabidiol
THC: Tetrahydrocannabinol
HL: Health literacy
IOC: Item-objective congruence
KR-20: Kuder-Richardson Formula 20
SD: Standard deviation
THB: Thai baht
